# DJ-1 promotes osteosarcoma progression through activating CDK4/RB/E2F1 signaling pathway

**DOI:** 10.3389/fonc.2022.1036401

**Published:** 2022-11-03

**Authors:** Zhitao Han, Lining Wang, Dongshuo Wang, Luosheng Zhang, Yifeng Bi, Xinlei Zheng, Weibo Liu, Guangjian Bai, Zhenhua Wang, Wei Wan, Yong Ma, Xiaopan Cai, Tielong Liu, Qi Jia

**Affiliations:** ^1^ School of Chinese Medicine, School of Integrated Chinese and Western Medicine, Nanjing University of Chinese Medicine, Nanjing, Jiangsu, China; ^2^ Laboratory of New Techniques of Restoration & Reconstruction, Institute of Traumatology & Orthopedics, Nanjing University of Chinese Medicine, Nanjing, Jiangsu, China; ^3^ Department of Orthopedic Oncology, Changzheng Hospital, Naval Medical University, Shanghai, China; ^4^ Department of Orthopaedics, the Fourth Medical Centre, Chinese PLA General Hospital, Beijing, China; ^5^ Department of Laboratory Medicine, Changzheng Hospital, Naval Medical University, Shanghai, China

**Keywords:** DJ-1 (PARK7), cdk4, RB, E2F1, osteosarcoma (OS), target therapy

## Abstract

Osteosarcoma (OS) is a primary malignant tumor of the bone characterized by poor prognosis due to chemotherapy resistance and high recurrence rates. DJ-1 (PARK7) is known as an oncogene and its abnormal expression is related to the poor prognosis of various types of malignant tumors. It was found in this study that upregulated expression of DJ-1 was closely correlated with the prognosis of OS patients by promoting the proliferation, migration and chemotherapy resistance of OS cells *in vitro* through regulating the activity of CDK4 but not through the oxidation mechanism or AKT pathway. The combination of DJ-1 and CDK4 promoted RB phosphorylation, leading to the dissociation of E2F1 into the nucleus to regulate the expression of cell cycle-related genes. The tumor xenograft mouse model demonstrated that DJ-1 knockout suppressed tumor growth *in vivo*. All these findings indicate that DJ-1 can affect the occurrence and progression of OS by regulating the CDK/RB/E2F1axis, suggesting a novel therapeutic opportunity for OS patients.

## Introduction

Osteosarcoma (OS) is an aggressive primary malignant tumor of the bone characterized by rapid progression, fast metastasis and high mortality, mainly affecting children, adolescents and young adults (1, 2). Although survival of OS patients has improved remarkably with the development of neo-adjuvant chemotherapy and surgical treatment techniques in recent years, the prognosis remains poor due to chemotherapy resistance and high recurrence rates (3). As the pathogenesis of OS is complex and there is a lack of understanding about the mechanism that drives the malignant properties of OS, it is essential to find the key genes that affect its development and prognosis for the sake of clarifying the pathogenesis of OS and providing evidence-based clues for developing new effective treatment regimens.

As a highly conserved homodimer protein encoded by PARK7 gene, DJ-1 was initially discovered by Nagakubo et al. (4), and later identified as a mitogen-dependent oncogene in Ras-related signaling pathway. Subsequently, PARK7 gene mutation was found as a causal factor for autosomal recessive early-onset Parkinson’s disease (AREP) (5). Currently, DJ-1 is recognized as a multifunctional protein participating in a variety of biological processes, including transcriptional regulation, anti-oxidative stress, and cellular transformation by acting as a redox-regulated molecular chaperone, cysteine protease, and transcriptional coactivator (6, 7).Multiple studies have shown that DJ-1 is overexpressed in many types of malignant tumors such as breast cancer, pancreatic cancer, and non-small cell lung cancer (NSCLC) (8–10). Its overexpression is found to be related to the proliferation, metastasis and poor prognosis of malignant tumors. Several molecular mechanisms of DJ-1 have been reported previously (9–11). Most importantly, as an endogenous antioxidant, DJ-1 regulates redox balance by activating Akt/mTOR, MEK/ERK, NF-κB and HIF-α signaling pathways, thereby enhancing the ability of tumor cells to resist oxidative stress (12, 13). Although there have been studies reporting the association between the expression of DJ-1 and osteosarcoma, the underlying molecular mechanism remains unclear (14).

The aim of this study was to evaluate the relationship between the expression of DJ-1 and the malignant biological behavior and chemotherapy resistance of OS, and further analyze the potential molecular mechanism. Our findings strongly suggest that DJ-1 functions as an oncogene in OS *via* CDK4/Rb/E2F1 signaling pathway. It is our hope that this new finding could help develop new targeted drugs for OS.

## Materials and methods

### Patients and tumor samples

We obtained 75 OS tissue samples with no chemotherapy or radiotherapy from the Human Tumor Tissue Biobank (Shanghai, China) between February 2013 and February 2018. The tissue samples were immediately snap-frozen and stored in liquid nitrogen for use. All human tissues were obtained with informed consent, and the project was approved by the Ethics Committee of Changzheng Hospital (Shanghai, China).

### Human cell culture

MG63, 143B, U2OS and Saos2 OS cell lines and human bone marrow mesenchymal stem cells obtained from Type Culture Collection (ATCC) were cultured in certain conditions as previously described (11).

### Immunohistochemistry and immunofluorescence staining assays

Sample processing was based on the standard procedure as previously described (13). The expression of DJ-I was detected by imaging with a microscope (Leica, Wetzlar, German) and measured with the antibodies against DJ1 (ab76008, Abcam, USA). The IHC stained specimens were assessed independently by three pathologists who were blind to the specimens. The staining intensity of positive cells was evaluated and their expressions were recorded. IF was quantitated with NIH Image J.

### Plasmid construction and siRNA

pcDNA3.1+ plasmid was digested by HindIII and Xho I (Takara, Japan). The code sequence of DJ-1 and CDK1-8 was amplified by RT-PCR and inserted into pcDNA3.1+ and pBiFC-VN173 respectively using the Quick-Fusion Cloning Kit (Biotool, USA). The RT-PCR primers are listed in **Supplementary Table 1**. Two different siRNA oligos against DJ-1 (siRNA-1: AAUUGUUGAAGCCCUGAAUCU; siRNA-2: UGGGAUUAAGGUCACCGUUCU) were purchased from Integrated DNA Technologies (IDT). Cells were transfected using Lipofectamine 3000 reagent and p3000 in OPTI-MEM reduced serum medium according to the instructions of the manufacturer. After transfection, the expression of DJ-1 was verified by Western blot.

### Western blotting analysis

Cells and clinical samples were lysed in RIPA buffer, and quantified by Rapid Gold BCA (Thermo, USA). Protein samples were separated equally by SDS-PAGE and electrotransferred to polyvinylidene difluoride (PVDF) membranes, which were then blocked for 1 h, and incubated with anti-DJ-1 (ab76008, Abcam, USA), anti-Bax (ab3191, Abcam, USA), anti-Bcl2 (ab196495, Abcam, USA), Anti-Cleaved Caspase-3(ab214430, Abcam, USA), Anti-CDK4(ab95255, Abcam, USA), Anti-Cyclin D1 (ab226977, Abcam, USA), Anti-E2F1 (ab137415, Abcam, USA), Anti-Cyclin D2 (ab230883, Abcam, USA), Anti-Cyclin D3 (ab112034, Abcam, USA), Anti-RB(17218-1-AP, proteintech, USA), Anti-pRb (phospho S780, ab47763,Abcam, USA), Anti-E2F1(12171-1-AP, proteintech, USA), Anti-H2A(16441-1-AP, proteintech, USA), Anti-α-Tubulin(11224-1-AP, proteintech, USA), Anti-Flag(80010-1-RR, proteintech, USA), and Anti-α-Actin(23660-1-AP, proteintech, USA). Next, the membranes were incubated in corresponding secondary antibodies for 1 h. Proteins were visualized and detected by SuperSignal West Atto(A38554, Thermo, USA).

### Cell proliferation and colony formation assays

Cells were seeded in 96-well plates to evaluate cell proliferation. After 48-h incubation, the procedures were conducted according to the Cell Counting Kit-8 (CCK-8) protocol. The optical absorbance (OD) was measured at 450 nm using the ELx800 microplate reader (BioTek Instruments Inc., USA). The colony formation assays were based on the standard procedure as previously described (13).

### Cell migration assays

Transwell migration assay was performed in the 8-μm pore transwell chamber without matrigel (3422, Corning, USA) according to the standard procedure as previously described (11).

### Flow cytometry analysis

Cell cycle distribution and apoptosis were determined by flow cytometry according to the standard procedure as previously described (13).

For cell apoptosis analysis, cells were seeded into a 6-well plate, treated with 0.4uM Adriamycin for 24 h, washed, and resuspended according to the kit’s instructions. The apoptosis rate was immediately analyzed after 15-min incubation at 25°C in dark.

### Co-immunoprecipitation assay

The protein extracts were prepared as described as Western blot and divided for Co-IP and input respectively. Quantitative protein lysates were incubated with a proper volume of Anti-CDK4, Anti-CDK4 and IgG antibody (ab172730, Abcam, USA) at 4°C overnight with gentle rotation. Then, the samples were incubated with magnetic beads (B23201, Bimake, USA) at room temperature for 2 h with gentle rotation. The immunoprecipitation products were centrifuged to remove the supernatant. The magnetic beads were washed with lysis buffer for at least three times repeatedly. 50 μl Loading Buffer was added into each tube and the samples were heated to 95°C for 5 min. The follow-up experiments are the same as Western blot.

### Quantitative real-time PCR

Sample processing was based on the standard procedure as previously described (13). The primer sequences are listed in **Supplementary Table 1**.

### Proximity ligation assay

293 cells in DJ-1 overexpression group and control group were seeded on fibronectin (10μg/mL)-coated chamber slides and permitted to attach overnight. The cells were processed using the manufacturers’ protocol of the Duolink *In Situ* Red Starter Kit and the signal was captured using Zeiss-LSM 510 confocal microscope.

### DJ-1 knockout cells

Target gDNA was designed by https://zlab.bio/guide-design-resources, and the selected AGTACAGTGTAGCCGTGATG (located on the 3 exon) was insert into lentiCRISPR v2. LentiCRISPR v2, pMD2.g and psPAX2 were co-transfected into 293T cells for 48 h to produce lentiviruses. The lentiviruses were harvested and used to infect MG63 cells for 48 h and purine was used for screening DJ-1 KO cells. Monoclonal cells were amplified and identified by DNA sequence and Western blot.

### The tumor xenograft mouse model

Nude mice aged 6 weeks were raised in specific-pathogen-free (SPF) conditions. All the animal experiments were approved by the Ethics Committee of the Naval Medical University (Shanghai, China).

The stable clones of DJ-1 control cells and DJ-1 KO cells were subcutaneously injected into the right upper flank of the 4-week-old male nude mice. The tumor volume was measured and calculated every other day. One month later, the tumors were dissected from the mice under anesthesia and weighed.

### Statistical analysis

Statistical analyses were conducted using SPSS 19.0 statistical software. All data are expressed as the mean ± standard error of the mean (SEM). The mean values between groups were assessed using both Student t test and ANOVA, assuming double-sided independent variance. *P <0.05, **P < 0.01, and ***P<0.001 were considered statistically significant.

## Results

### High DJ-1 expression is correlated with poor survival of OS patients

**Figure 1:**
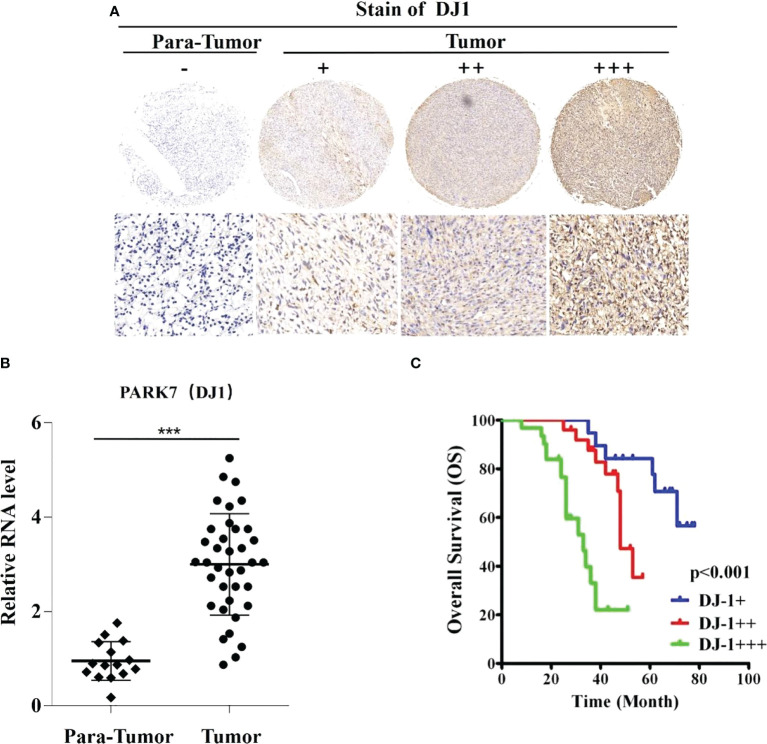
DJ-1 expression is associated with poor survival in osteosarcoma (OS). Representative immunohistochemical staining of DJ-1 in human OS tissues (n=75) and negative control. **(A)** The nuclear staining of DJ-1 was categorized and represented as follows: score 1(+), score 2(++), score 3(+++). **(B)** Relative RNA level in OS lesions was higher than that in para-tumor tissues. **(C)** The overall survival curves of OS patients (n=75) are shown for different DJ-1 expression levels. Log-rank tests were used to determine statistical significance. ***P < 0.001.

The correlation between DJ-1 expression and survival of OS patients was investigated (**Figure 1**). The protein expression levels and mRNA levels of DJ-1 were analyzed with IHC staining and qRT-PCR in the 75 OS samples and para-tumor samples (**Figures 1A, B**). According to the nuclear staining score, DJ-1 expression was grouped as high (3 points), intermediate (2 point) and low (1 point).

DJ-1 expression level was found high, intermediate and low in 41.3% (31/75), 33.3% (25/75), and 25.3% (19/75) tissues, respectively. Notably, DJ-1 expression level was closely correlated with the overall survival of the OS patients (P<0.001) (**Figure 1C**). In addition, correlation analysis showed that DJ-1 expression was positively correlated with tumor size (P=.013), recurrence (P=.009) and metastasis (P=.033) (**Table 1**).

**Table 1:** Correlation of DJ-1 expression with clinical features of patients with osteosarcoma.

Features	No.	DJ-1 expression			χ²	P
		+++	++	+		
**Gender**						
male	43	18	14	11	0.027	0.986
female	32	13	11	8
**Age**						
≤20	46	17	16	13	1.029	0.598
>20	29	14	9	6
**Enneking stage**						
I	14	5	4	5	1.552	0.817
II	36	16	13	7
III	25	10	8	7
**Histologic type**						
Osteoblastic	43	18	12	13	3.265	0.515
Osteolytic	16	7	5	4
Mixed	16	6	8	2
**Location**						
spine	55	23	19	13	0.337	0.845
extremities	20	8	6	6
**Tumor size**						
Large (>5 cm)	41	23	9	9	8.693	0.013
Small (≤5 cm)	34	8	16	10
**Recurrence**						
Presence	24	16	4	4	9.468	0.009
Absence	51	15	21	15
**Tumor metastasis**						
Presence	20	13	5	2	6.795	0.033
Absence	55	18	20	17

### Increased expression promotes OS cell proliferation, clonal formation and migration

**Figure 2:**
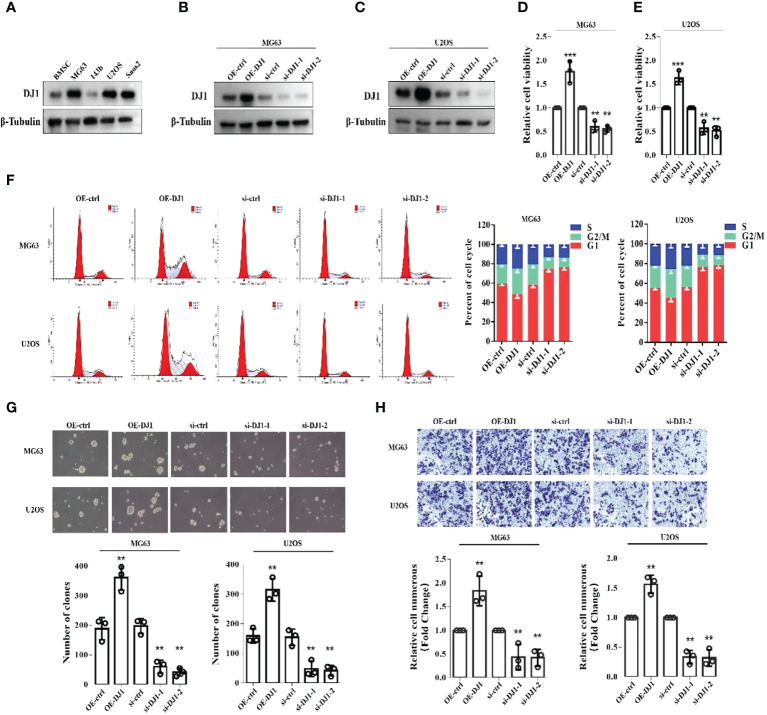
Increased expression of DJ-1 in OS cell lines promotes cell proliferation, clonal formation and migration. **(A)**. WB of DJ-1 expression on a panel of human BMSCs and OS cell lines; **(B, C)**. Overexpression or knockdown of DJ-1 in MG63 and U2OS cell lines were constructed. **(D, E)**. CCK-8 was performed to observe the effects of DJ-1 knockdown or overexpression on cell proliferation in MG63 and U2OS cells. **(F)**. Cell cycle distribution was evaluated by flow cytometry and shown by bar charts. **(G, H)**. Colony formation and transwell migration assays indicated that DJ-1 could promote the migration of OS cells. The Original uncut western bands are shown in **Supplementary Figure 3**. n = 3 per group. **P < 0.01; ***P < 0.001.

To clarify whether DJ-1 played a role in the pathophysiology of OS, we first detected the expression level of DJ-1 in bone mesenchymal stem cells (BMSCs) and four OS cell lines (MG63, 143B, U2OS and Saos2) and found that DJ-1 expression level in MG63\U2OS\SAOS2 OS cells was higher than that in BMSCs (**Figure 2A**). Subsequently, we constructed DJ-1 knockdown and overexpression cell lines in MG63 and U2OS cell lines, and verified the successful construction by Western Blot (**Figures 2B, C**). The CCK8 assay showed that overexpression of DJ-1 increased the proliferation of OS cells, while knockdown inhibited their proliferation (**Figures 2D, E**). Cell cycle analysis by flow cytometry indicated that DJ-1 overexpression significantly increased the ratio of G2-phase cells, which could accelerate cell cycle and promote cell proliferation (**Figure 2F**). Colony formation and transwell migration assays indicated that DJ-1 overexpression significantly increased the number of clones and invading OS cells, while knocking down DJ-1 reduced the number of clones and invading OS cells accordingly, suggesting that DJ-1 could promote the migration and invasion of OS cells (**Figures 2G, H**). These results indicate that increased expression of DJ-1 in OS cell lines promotes cell proliferation, clonal formation and migration.

### DJ-1 mediates the resistance of OS cells to Adriamycin-induced apoptosis

**Figure 3:**
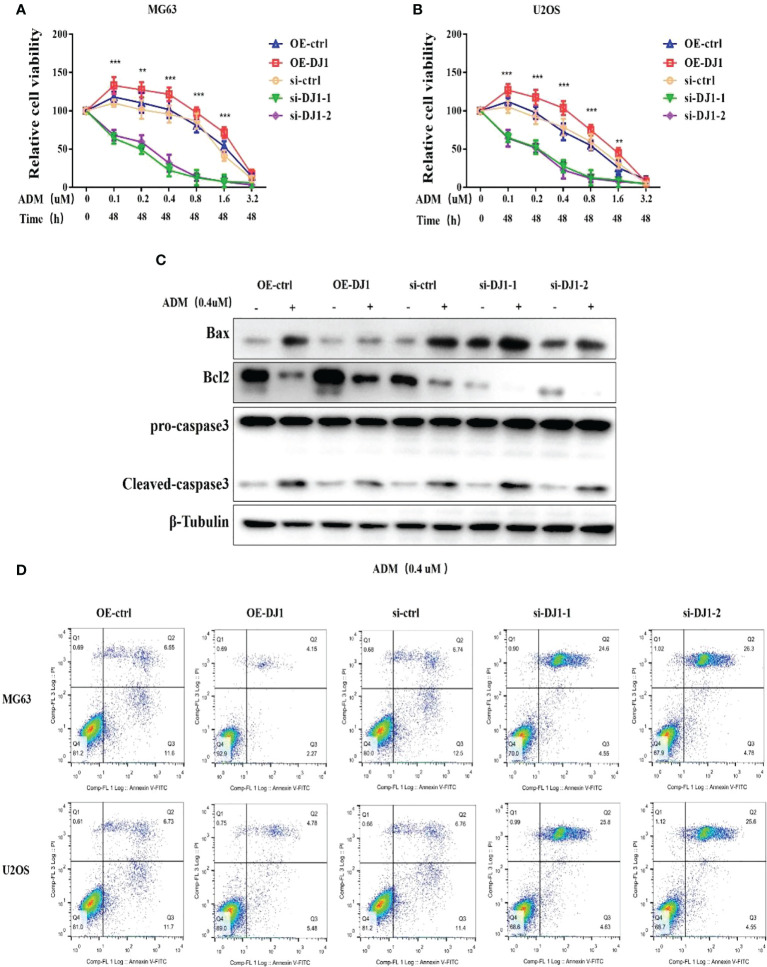
DJ-1 mediates the resistance of OS cells to Adriamycin-induced apoptosis. **(A, B)**. After 24-h treatment with different concentrations of Adriamycin, the viabilities of MG63 and U2OS cells with DJ-1 overexpression or knockdown was detected and compared. **(C)**. WB results indicated that up-regulation of DJ-1 could reduce the expression of Bax and cleave caspase-3 activity in OS cells. **(D)**. Annexin V/PI double-staining assay showed that DJ-1 down-regulation aggravated OS cell apoptosis. The Original uncut western bands are shown in **Supplementary Figure 3**. n = 3 per group. **P < 0.01; ***P < 0.001.

Adriamycin is a common anti-tumor drug which can inhibit RNA and DNA synthesis. Because of its broad anti-tumor spectrum, Adriamycin is generally used as a first-line drug for OS. Although several signaling pathways have been reported to be related to Adriamycin resistance, its mechanism remains unclear (15). To assess the role of DJ-1 in Adriamycin resistance of OS cells, MG63 and U2OS cells with DJ-1 overexpression or knockdown were treated with different concentrations of Adriamycin. The CCK8 assay results demonstrated that OS cell proliferation was significantly inhibited in a dose-dependent manner, and DJ-1 knockdown increased the sensitivity of OS cells to Adriamycin. On the contrary, DJ-1 overexpression led to resistance to Adriamycin (**Figures 3A, B**). In order to detect the expression level of apoptosis-related proteins, MG63 cells were transfected with DJ-1 over-expression plasmid or siRNA for 24 hours, and were then treated with Adriamycin (0.4uM) for 24 hours. We found that the up-regulation of DJ-1 significantly reduced the expression of Bax mediated by Adriamycin compared with the control cells, thereby reducing caspase-3 activity in OS cells and inhibiting cell apoptosis (**Figure 3C**). Consistent with this finding, the Annexin V/PI double-staining assay demonstrated that DJ-1 down-regulation aggravated OS cell apoptosis induced by Adriamycin (**Figure 3D**). These findings indicate that DJ-1 could mediate the resistance of OS to Adriamycin by affecting the proliferation and apoptosis.

### DJ-1 interacts with cyclin-dependent kinases 4

**Figure 4:**
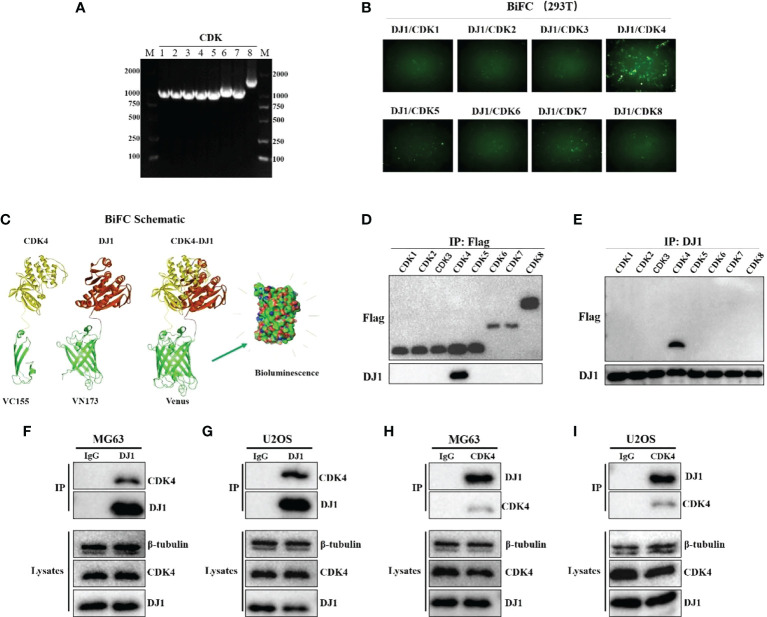
DJ-1 interacts with CDK 4. **(A)**. CDS of CDK1-8 agarose gel electrophoresis. **(B)** DJ-1 and CDK plasmids with GFP tag were co-transfected in 293 cells, and the protein interaction could be revealed by the specific complementation of the fluorescent probe and the resulting green fluorescence emission. **(C)** the BiFC schematic of CDK4 and DJ1. **(D, E)**. The CDK plasmids with Flag tag were transfected in 293 cells, and the interaction of CDK4 with DJ-1 was confirmed by Co-IP. Exogenous DJ-1 with flag was expressed in 293 cells and found to be interacted with CDK4. **(F-I)**. The pull-down results of endogenous DJ-1 and endogenous CDK4 in MG63 and U2OS cells were confirmed by Co-IP. The Original uncut western bands are shown in **Supplementary Figure 4**.

The oxidation state of DJ-1 is significant to the anti-oxidative stress process and cell protection, and the 106th cysteine of DJ-1 is an important oxidation target (16). However, DJ-1 C106A mutation did not hinder the OS cell proliferation, migration and invasion (**Supplementary Figure 1**). It was reported that high expression of DJ-1 was involved in the activation of PI3K-Akt signaling pathway (15), but AKT inhibitor did not significantly inhibit OS cell proliferation (**Supplementary Figure 2**). Cell cycle disruption is one of the biomolecular mechanisms of OS (17). Cell cycle analysis by flow cytometry indicated that DJ-1 overexpression significantly increased the ratio of G2-phase cells, which could accelerate cell cycle and promote cell proliferation. Cyclin-dependent kinase (CDK) is an important factor in cell cycle regulation. The activity of CDK can be regulated by certain oncogenes and tumor suppressor genes (18), but whether DJ-1 mediated the malignant biological behavior of OS cells by regulating the activity of CDK remained unclear. To answer this question, we first used the bimolecular fluorescent complimentary (BiFC) to determine whether there was an interaction between DJ-1 and CDK. The GFP complementation in 293 cells overexpressing DJ-1 and CDKs revealed that DJ-1 was able to bind CDK4 (**Figures 4A-C**). Then, the exogenous CDK 1-8 with flag was expressed in 293 cells, and the interaction of CDK4 with DJ-1 was confirmed by Co-IP. Similarly, exogenous DJ-1 with flag was expressed in 293 cells and was found to be interacted with CDK4 (**Figures 4D, E**). Furthermore, the binding of endogenous DJ-1 and endogenous CDK4 in MG63 and U2OS cells were confirmed by Co-IP (**Figures 4F-I**).

### DJ-1 activates the Rb/E2F1/DP1 pathway through CDK4

**Figure 5:**
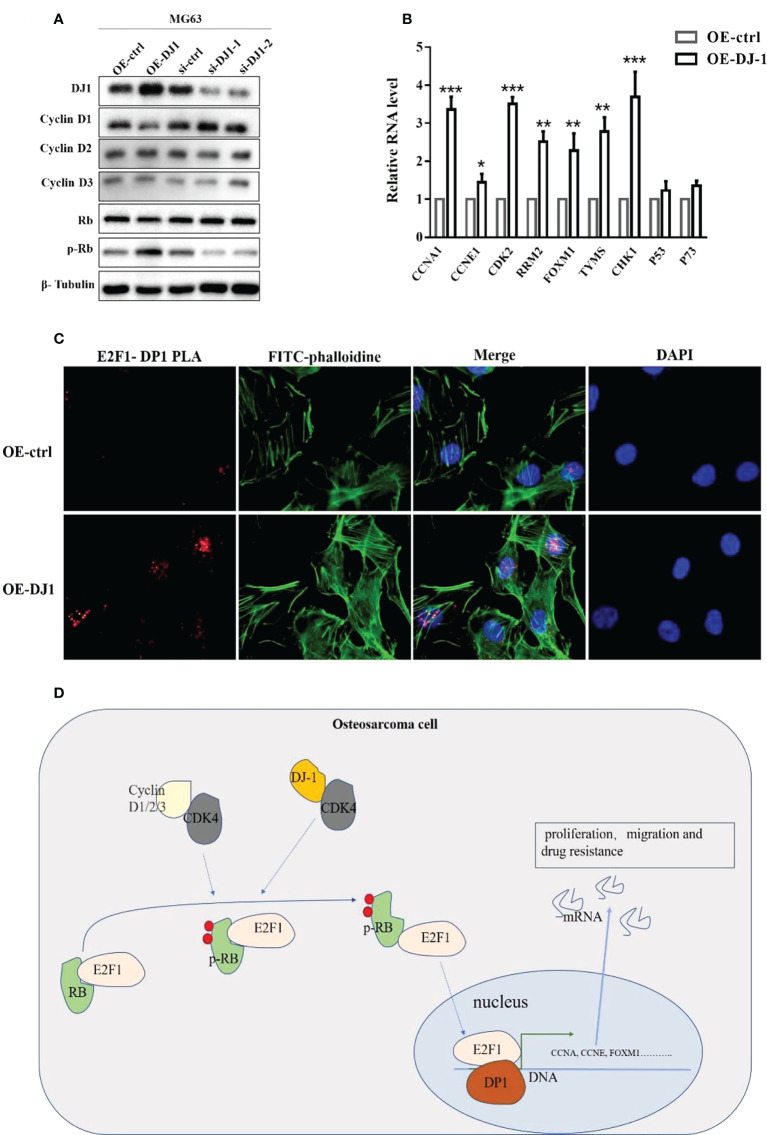
DJ-1 activates the Rb/E2F1/DP1 pathway through CDK4. **(A)** WB of cyclin D1, cyclin D2, cyclin D3, Rb and p-Rb expressions on MG-63 cells with DJ-1 overexpression or knockdown. **(B)** The mRNA levels of E2F1 downstream genes CCNA, CCNE, CDK2, RRM2, FOXM1, TVMS, CHK1, P53, and P73. **(C)** PLA analysis showed that overexpression of DJ-1 could increase the binding degree of E2F1/DP1 in the nucleus. **(D)** Schematic illustrating the potential role of DJ-1 in OS occurrence and progression. The original uncut western bands are shown in **Supplementary Figure 4**. n =3 per group. *P < 0.05; **P < 0.01; ***P < 0.001.

To further study the effect of DJ-1/CDK4 interactions, we investigated the phosphorylation level of retinoblastoma (Rb) protein, knowing that Rb is a key effector downstream of CDK4/cyclin D, which is usually in a low phosphorylation state and binds to the transcription factor E2F1 to inhibit the cell cycle (19, 20). Western blot results showed that overexpression of DJ-1 could promote Rb phosphorylation with no influence on the expression level of Cyclin 1-3 (**Figure 5A**), which led to the increased expression of cell cycle-promoting genes (CCNA, CCNE, CDK2, RRM2, FOXM1, TVMS and CHK1) (**Figure 5B**). The transcription factor DP1 was initially identified as the binding partner for E2F1, and E2F1/DP1 played a crucial role in coordinating gene expression during G1/S cell cycle progression (20).

To explore whether phosphorylated Pb could release E2F1 into the nucleus, PLA analysis was conducted to investigate the binding level of E2F1 and DP1 after DJ1 overexpression. The results showed the binding degree of E2F1/DP1 was increased in the nucleus, suggesting that DJ-1 could regulate Rb phosphorylation by interacting with CDK 4 and release E2F1 into the nucleus to regulate the malignant behavior of cells by binding with DP1 (**Figures 5C, D**).

### Knockout of DJ-1 suppresses tumor growth *in vivo*

**Figure 6:**
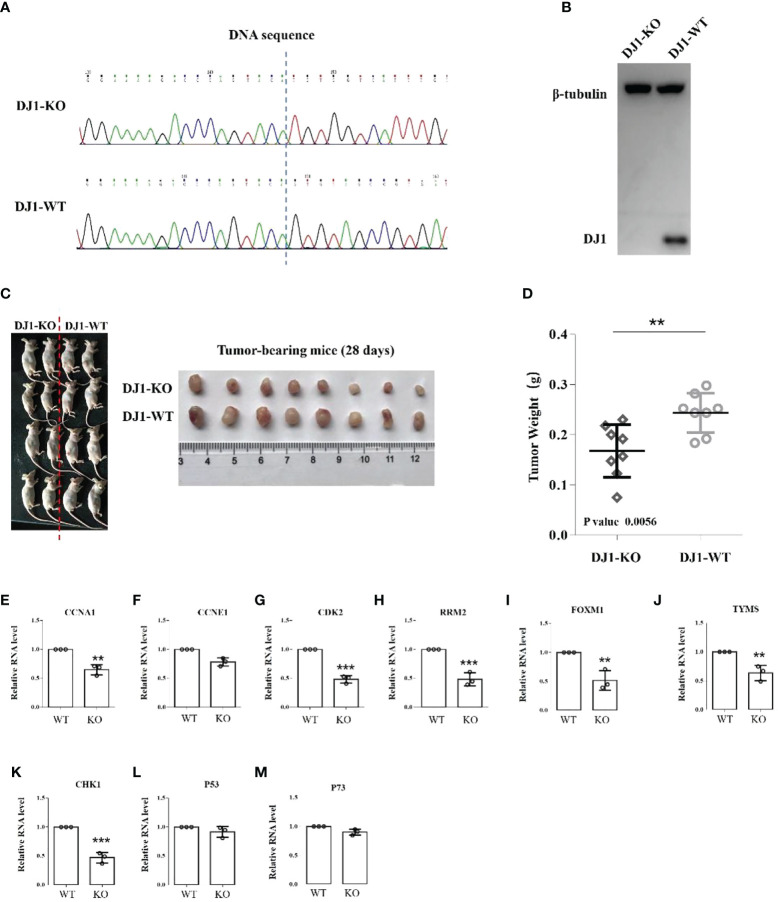
Knockout of DJ-1 suppresses tumor growth *in vivo*. **(A, B)**. DNA sequencing and Western Blot verified that DJ-1 was knocked out in MG63 cells. **(C, D)**. MG63 DJ-1 WT and DJ-1 KO cells were subcutaneously injected into nude mice and tumor weight was recorded. **(E–M)**. RNA expression of CCNA1, CCNE1, CDK2, RRM2, FOXM1, TVMS, CHK1, p53 and p73. The original uncut western bands are shown in **Supplementary Figure 4**. **P < 0.01; ***P < 0.001.

To further determine whether the carcinogenic and aggressive properties could be reversed by eliminating DJ-1 expression, the capability of tumor cell growth *in vivo* was evaluated by xenograft tumor assay. We constructed a DJ-1 KO clone in MG63 cells and verified the knockout efficiency by Western Blot and sequencing (**Figures 6A, B**). DJ-1 WT and DJ-1 KO cells were subcutaneously injected into nude mice. It was found that tumors formed by DJ-1 KO cells were significantly lighter than those formed by DJ-1 WT cells (p<0.01) (**Figures 6C, D**). Furthermore, we detected the mRNA expression of cell cycle-promoting genes in tumor-bearing tissues, and found that DJ-1 KO decreased the expression of cell cycle-promoting genes (CCNA1, CCNE1, CDK2, RRM2, FOXM1, TVMS and CHK1) (**Figures 6C-K**), but the expression of p53 and p73 remained unchanged significantly (**Figures 6L, M**). These results implied that DJ-1 KO attenuated tumor growth *in vivo* through affecting the cell cycle of OS cells.

## Discussion

Osteosarcoma is a common malignant primary bone tumor, often involving the epiphysis of the long shaft and occasionally the spine. Although the 5-year survival rate of OS patients has increased to 50% after receiving surgery and chemotherapy in the past 30 years, this rate has not been further improved due to resistance to traditional chemotherapeutic drugs. Therefore, special attention should be paid to reveal the molecular mechanism of the occurrence and progression of OS for the sake of improving the individualized therapeutic effectiveness. DJ-1 is a novel oncogene with an important transforming activity, and plays vital roles in tumor occurrence, progression, metastasis, oxidative stress and apoptosis (17, 21). However, its role in OS and potential molecular mechanism have been rarely reported and poorly understood. Our experimental results demonstrated that DJ-1 expression was correlated with poor survival of OS patients, and high expression of DJ-1 increased the resistance ability to Adriamycin. DJ-1 knockdown arrested the cell cycle of OS cells, increased apoptosis and weakened tumorigenicity. These results *in vivo* and *in vitro* suggested that DJ-1 affected the malignant behaviors of OS *via* CDK4/Rb/E2F1 signaling pathway.

DJ-1 could participate in tumor occurrence, progression and drug resistance through a variety of mechanisms. For example, DJ-1 can inhibit PTEN, thereby activating PKB/Akt signaling, leading to the progression of lung cancer, breast cancer, and clear cell renal cell carcinoma (8–10). While in pancreatic cancer, DJ-1 promotes cell migration and invasion by activating ERK pathway. And DJ-1 could promote colorectal cancer metastasis by activating PLAGL2-Wnt-BMP4 axis (11). To identify the molecular signal cascade of DJ-1 involved in the development and progression of OS, we first investigated whether the oxidation state of DJ-1 played any significant role in the malignant progression of OS cells. DJ-1 contains three cysteine residues (C46, C56 and C106), among which C106 is highly susceptible to oxidative stress (16, 22, 23). We therefore constructed a DJ-1 C106A mutant and found that DJ-1 C106A mutation had no significant impact on the proliferation, invasion and Adriamycin resistance of OS cells as compared with DJ-1 WT. This indicates that DJ-1 does not maintain the survival of OS cells through the oxidative stress defense mechanism.

Cell proliferation is an important mechanism of eukaryotic growth, progression and regeneration. However, it is also the cause of cancer. Cell proliferation depends on the progression of the four different stages of the cell cycle (G0/G1, S, G2, and M). Disruption of cell cycle regulation in tumor cells leads to an uncontrolled proliferation state (24). Multiple CDKs cooperate with cyclins to drive the progress of each phase of the cell cycle (25). Two cell cycle kinase complexes (CDK4/6-cyclin D and CDK2-cyclin E) are important regulatory molecules for the G1-S phase checkpoint (26). Here we found that DJ-1 could directly bind to CDK4 but had no interaction with CDK2 and CDK6, indicating that DJ-1 may regulate the G1/S transition through regulation of CDK4. Furthermore, our experiments demonstrated that DJ-1 could phosphorylate the downstream effector Rb, leading to the release of E2F1 transcription factor into the nucleus without promoting the expression of cyclin D. The transcriptional activation of S-phase related genes is only one aspect of E2F1 activity. It can also regulate the expression of pro-apoptotic genes (p53 and p73) to mediate cell apoptosis (27, 28). However, the transcription level of the two important tumor suppressor genes p53 and p73 did not increase accordingly when DJ-1 was overexpressed, which means that DJ-1 promoted the apoptosis not through the regulation of p53 and p73. The mechanism by which E2F1 regulates the balance of proliferation and apoptosis in osteosarcoma cells remains unclear. It may be related to the expression profile in tumor cells, suggesting that E2F1 may have a selective effect in different cell types.

Cancer is pathologically characterized by uncontrolled cell division and therefore a better understanding about the basic principles of cell cycle control would help develop effective cancer therapies (29, 30). CDKs play a key role in cell cycle regulation and therefore may prove to the key therapeutic target. Some studies demonstrated that many tumorigenic events ultimately drove cancer cell proliferation by regulating CDK4 or CDK6 complexes in the G1 phase of cell cycle. The results of our experiments suggest the potential of using CDK4 inhibitors as the therapeutic target of OS in future (31).

In conclusion, our study demonstrated that DJ-1 could promote the proliferation, migration and chemotherapy resistance of OS cells, and that high expression of DJ-1 was associated with poor prognosis of OS patients. In addition, we revealed a novel molecular mechanism of DJ-I on OS by activating CDK4/Rb/E2F1 signaling pathway. This signaling pathway may prove to be a target for designing novel therapeutic strategies for OS.

## Data availability statement

The original contributions presented in the study are included in the article/**Supplementary Material**. Further inquiries can be directed to the corresponding authors.

## Ethics statement

The studies involving human participants were reviewed and approved by Shanghai Changzheng Hospital. The patients/participants provided their written informed consent to participate in this study. The animal study was reviewed and approved by Shanghai Changzheng Hospital. Written informed consent was obtained from the owners for the participation of their animals in this study.

## Author contributions

QJ, ZTH, and TLL contributed to conception and design of the study. LNW and DSW organized the database. DSW, LSZ, YFB, XLZ, WBL, GJB and ZHW performed the experiments. QJ and YM performed the statistical analysis. QJ, ZTH and DSW wrote the first draft of the manuscript. QJ and ZTH wrote sections of the manuscript. All authors contributed to the article and approved the submitted version.

## Funding

The work is supported by the National Natural Science Foundation of China (82002838, 81972506, 81871470), Sailing Talent Program of Naval Medical University (SL17), and Shanghai Youth Science and Technology Talent Sailing Program (20YF1449100). Project of Shanghai Municipal Commission of Science and Technology (19411962700).

## Conflict of interest

The authors declare that the research was conducted in the absence of any commercial or financial relationships that could be construed as a potential conflict of interest.

## Publisher’s note

All claims expressed in this article are solely those of the authors and do not necessarily represent those of their affiliated organizations, or those of the publisher, the editors and the reviewers. Any product that may be evaluated in this article, or claim that may be made by its manufacturer, is not guaranteed or endorsed by the publisher.
