# Supplementary material for: DJ-1 promotes osteosarcoma progression through activating CDK4/RB/E2F1 signaling pathway
Source: Front Oncol. 2022 Nov 3;12:1036401. doi: 10.3389/fonc.2022.1036401 (PMC9671360; doi:10.3389/fonc.2022.1036401)

Supplementary Figure. 1 The tumor-promoting effect of DJ-1 is not through the anti-oxidative stress pathway. A. The 106th amino acid of DJ-1 was mutated from cysteine to alanine. B-D. Comparison of proliferation, migration and resistance to Adriamycin in control cells, DJ-1 cells and DJ-1 C106A cells by CCK8 assay, transwell migration assay and Annexin V/PI double-staining assay, respectively.

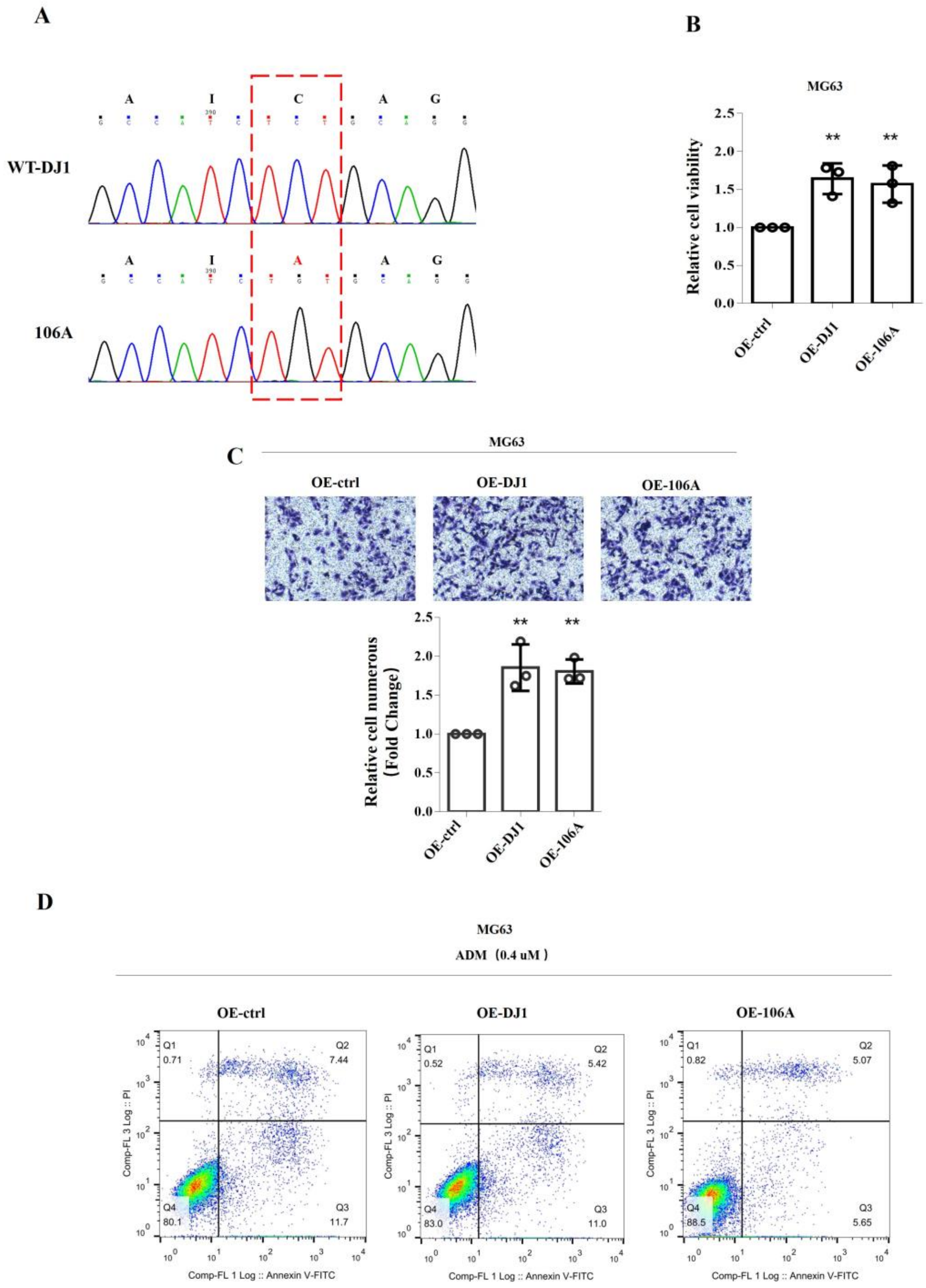

Supplementary Figure. 2 The tumor-promoting effect of DJ-1 independent of AKT signaling pathway. A-B: CCK-8 and Annexin V/PI double-staining assay was performed to observe the effects of DJ-1 when AKT inhibitor presence.

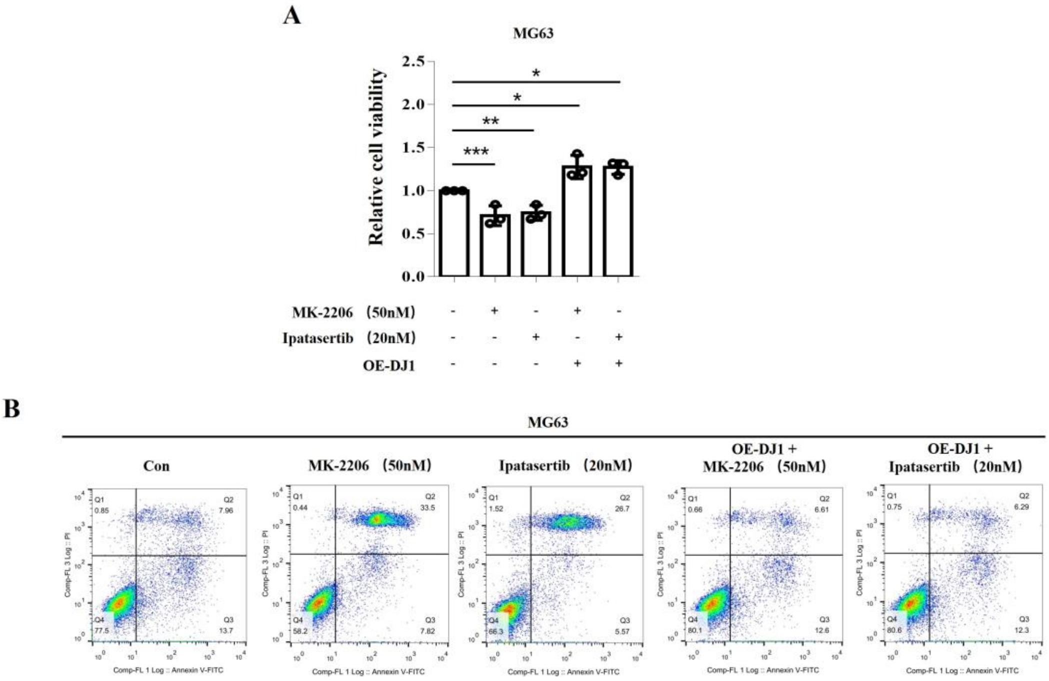

Supplementary Figure. 3 The Original uncut western bands of Figure 2A, 2B, 2C, and 3C.

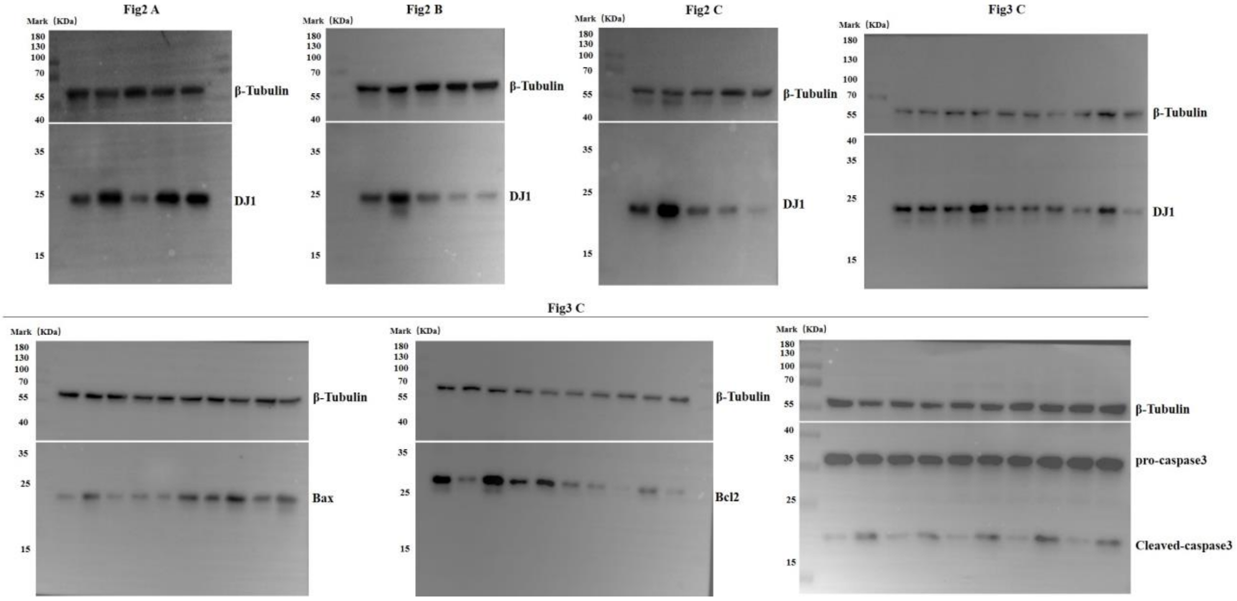

Supplementary Figure. 4 The Original uncut western bands of Figure 4D-I, 5A and 6B

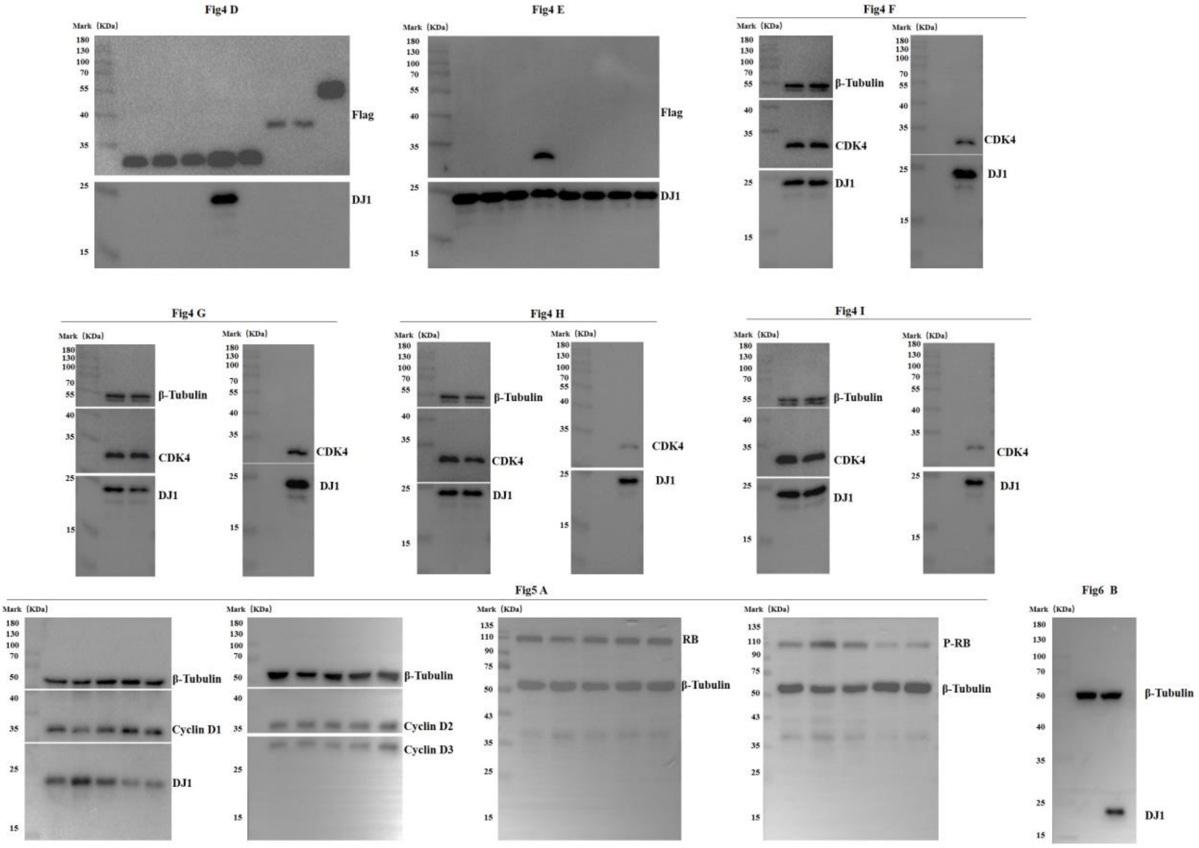

Supplement: Supplementary file 1 [file DataSheet_1.pdf]
