# Supplementary material for: DJ-1 promotes osteosarcoma progression through activating CDK4/RB/E2F1 signaling pathway
Source: Front Oncol. 2022 Nov 3;12:1036401. doi: 10.3389/fonc.2022.1036401 (PMC9671360; doi:10.3389/fonc.2022.1036401)
Supplement: Supplementary file 2 [file Table_1.doc]

| [**Supplementary**](javascript:;) **table 1** Primer for q-RTPCR | | |
| --- | --- | --- |
| Name | Forward primer | Reverse primer |
| CDK1 | ATGGAAGATTATACCAAAATA | CATCTTCTTAATCTGATTGTCCAA |
| CDK2 | ATGGAGAACTTCCAAAAGGTGG | GAGTCGAAGATGGGGTACTGGCTT |
| CDK3 | ATGGATATGTTCCAGAAGGTA | ATGGCGGAATCGCTGCAGCAC |
| CDK4 | ATGGCTACCTCTCGATATGAGCC | CTCCGGATTACCTTCATCCTTAT |
| CDK5 | ATGCAGAAATACGAGAAACTG | GGGCGGACAGAAGTCGGAGAAGTA |
| CDK6 | ATGGAGAAGGACGGCCTGTG | GGCTGTATTCAGCTCCGAGGTGT |
| CDK7 | ATGGCTCTGGACGTGAAGTCTCG | AAAAATTAGTTTCTTGGGCAATCCT |
| CDK8 | ATGGACTATGACTTTAAAGTGAA | GTACCGATGTGTCTGATGTGAG |
| Park7 | GCAGAGGAAATGGAGACG | AAGGCTGGCATCAGGAC |
| CCNA1 | GGTCCAGGCAGGTTTTG | GGTCTCCATCCCAAGTGA |
| CCNE1 | GCCACAGAGCGGTAAGAA | CAGAAGAGAACGTGGAGCA |
| CDK2 | TGCTGAGATGGTGACTCG | ACTTGGGGAAACTTGGCT |
| RRM2 | GAAAACTTGGTGGAGCGA | GCGGGCTTCTGTAATCTG |
| FOXM1 | GCCTCCCTGTGTGGATG | ATGGGCAGCGTTTCCTT |
| TYMS | AGTGGAGGCATTTTGGG | GGGTTGGTTTTGATGGTG |
| CHEK1 | AACCAAGTTTCAGGGGACA | TGGCTTCGCTTCACAGA |
| P53 | CCAGATGAAGCTCCCAGA | GGGAAGGGACAGAAGATGA |
| P73 | AAGCAGCGACTGGTGTTG | ACGGTGGGAGAGGGATG |
